# Regenerated silk fibroin based on small aperture scaffolds and marginal sealing hydrogel for osteochondral defect repair

**DOI:** 10.1186/s40824-023-00370-1

**Published:** 2023-05-19

**Authors:** Yinyue Luo, Menglin Xiao, Bushra sufyan Almaqrami, Hong Kang, Zhengzhong Shao, Xin Chen, Ying Zhang

**Affiliations:** 1 Department of Preventive Dentistry, Shanghai Stomatological Hospital & School of Stomatology, Department of Macromolecular Science, Fudan University, Shanghai, 200001 China; 2 Shanghai Key Laboratory of Craniomaxillofacial Development and Diseases, Fudan University, Shanghai, 200002 China; 3 State Key Laboratory of Molecular Engineering of Polymers, Laboratory of Advanced Materials, Fudan University, Shanghai, 200433 China; 4 Department of Orthodontics, Ningbo Dental Hospital, Ningbo, 315032 Zhejiang China; 5 Department of Temporomandibular Joint and Occlusion, School/Hospital of Stomatology, Lanzhou University, Lanzhou, Gansu, 730013 China

**Keywords:** *n*-butanol, Small aperture scaffolds, Regenerated silk fibroin hydrogel, Osteochondral defects

## Abstract

**Background:**

Osteochondral defects pose an enormous challenge without satisfactory repair strategy to date. In particular, the lateral integration of neo-cartilage into the surrounding native cartilage is a difficult and inadequately addressed problem determining tissue repair’s success.

**Methods:**

Regenerated silk fibroin (RSF) based on small aperture scaffolds was prepared with *n*-butanol innovatively. Then, the rabbit knee chondrocytes and bone mesenchymal stem cells (BMSCs) were cultured on RSF scaffolds, and after induction of chondrogenic differentiation, cell-scaffold complexes strengthened by a 14 wt% RSF solution were prepared for in vivo experiments.

**Results:**

A porous scaffold and an RSF sealant exhibiting biocompatibility and excellent adhesive properties are developed and confirmed to promote chondrocyte migration and differentiation. Thus, osteochondral repair and superior horizontal integration are achieved in vivo with this composite.

**Conclusions:**

Overall, the new approach of marginal sealing around the RSF scaffolds exhibits preeminent repair results, confirming the ability of this novel graft to facilitate simultaneous regeneration of cartilage–subchondral bone.

**Supplementary Information:**

The online version contains supplementary material available at 10.1186/s40824-023-00370-1.

## Introduction

Articular cartilage covers the ends of the bones where they meet to form joints, providing a smooth, lubricated surface that allows low-friction movement [1]. The articular cartilage is avascular and aneural, that is, it lacks blood vessels and nerves [2]. Therefore, it cannot regenerate itself when damaged [3]. Various novel solutions have been developed to mitigate this deficiency, among which tissue-engineered cartilage offers a unique opportunity to address this conundrum [4]. The scaffold-based tissue engineering approach results in better functional outcomes, improved cartilage development, and fewer adverse events. However, it suffers from lack of integration, loss of cell viability due to graft storage, and potential disease transmission [5]. Despite advances in cartilage tissue engineering, many challenges remain in recreating a complete functional organ that is integral, viable, and disease-free by stacking biological elements within a 3D structure. This complex process has yet to include optimizations of the aperture for chondrocyte proliferation, lateral integration of the cartilage implant and native tissue, and long-term follow-up after in vivo implantation.

Silk fibroin (SF), a nonbioactive protein derived from Bombyx mori silkworm silk, is widely used as a biomaterial. Various forms, such as scaffolds, powders, membranes, films, and hydrogels, have been used for biomedical applications, including artificial skin, auricular cartilage, and bone, thereby attracting considerable interest in the development of tissue engineering recently [6]. Furthermore, the chemical and physical properties of the SF scaffolds can be tailored by controlling their nanostructure and porosity to satisfy the specific requirements for tissue engineering [7]. In addition, SF-based hydrogels have been used for tissue engineering applications as a 3D cell culture system, due to their highwater content and flexibility. Cellular biological behavior is guided by biomimetic scaffolds for tissue/organ regeneration [8]. Scaffolds are designed to mimic the extracellular matrix (ECM) of native tissues, and their pore size can significantly affect the ability of cells to migrate, proliferate, and produce ECM components. Smaller pore sizes allow greater control over the microenvironment of the scaffold, whereas larger pore sizes allow greater cell infiltration and access to the scaffold surface [9]. This condition can influence cell–cell interactions, as well as the ability of growth factors to bind to the surface of the scaffold. In addition, the pore size of the scaffolds affects the diffusion of oxygen and nutrients, which is necessary for cell growth and survival [10]. Pore sizes inspire us to create biomimetic scaffolds to investigate the effects of the aperture network on multiple cell events. However, the vintage pore size of the material suitable for chondrocytes to live and integrate with the host tissue remains debatable.

Several factors impede cartilage lateral integration. First, the ECM has low metabolism, is avascular, 3D dense, and anti-adhesive [11]. For example, glycosaminoglycans (GAGs) have been proven to directly hinder cell adhesion [12]. Second, cell death, as a result of surgical preparation at the defect edges, reduces integration potential [13]. Third, stress concentration occurs when the biomechanical properties of the implants and native tissues are mismatched under forces, damaging the surrounding tissue and weakening integration [14]. Strategies for enhancing lateral integration include anti-apoptosis agents [13], matrix-degrading enzymes [11], and, more recently, scaffold functionalization to enable direct bonding to adjacent cartilage [15]. However, these studies were largely conducted either in vitro or in murine subcutaneous implantation mode and rarely validated with effects on osteochondral repair in large animals. Lateral integration remains a significant unsolved problem because the safety and efficacy of these treatments have yet to be determined. Lateral integration remains a significant problem because its safety and efficacy have yet to be determined. One potential solution is using hydrogels, which can serve as buffers between implants and local tissues because of their viscosity and gelatinous properties. In situ cross-linked carboxymethyl chitosan/oxidized dextran/poly-γ-glutamic acid hydrogels significantly inhibit bacterial growth and promote wound healing [16]. A dual-network hydrogel composed of sodium alginate and platelet-rich plasma applied to the wound skin of rats was highly effective in closing the wound [17]. Hydrogels are competitive because of their adhesive properties, synergistic therapeutic effects, pH sensitivity, temperature sensitivity, and size-targeting features [18]. A high-concentration (> 12 wt%) RSF solution has high shear sensitivity and can quickly form a hydrogel due to shear-induced β-sheet formation [19]. The flexion, extension, and rotation of the knee joint provide a natural environment for β-sheet formation.

**Scheme 1:**
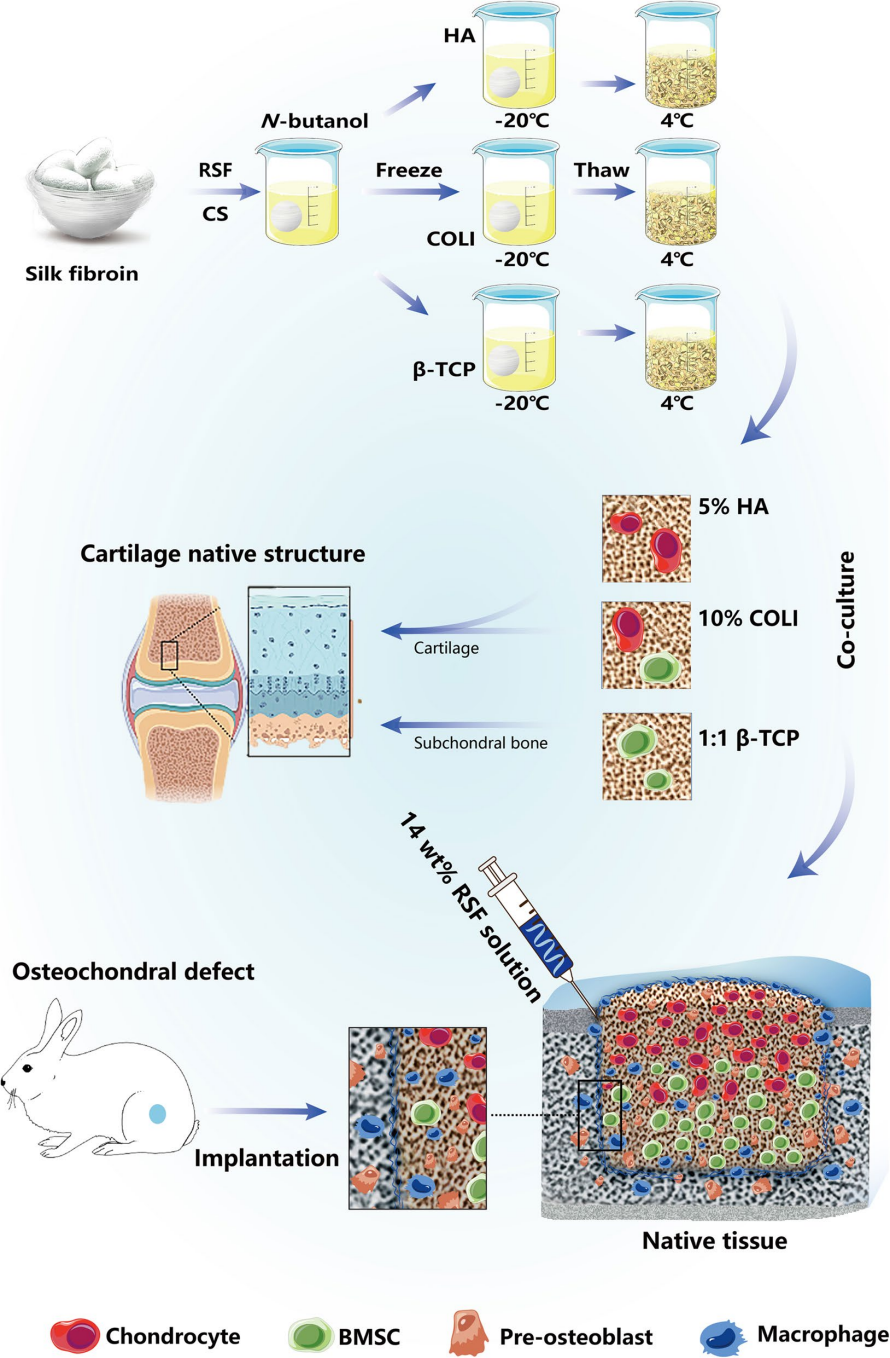
A schematic of the synthesis process and therapeutic effects of a marginal sealant and RSF scaffold based on *n*-butanol for the repair of osteochondral defects is presented. The RSF scaffold is synthesized by a two-step pyrolysis-leaching method using *n*-butanol as porogen, in which hierarchical mesoporous structures with abundant ECM sites are formed. The degummed silk is initially assembled with 3.15 wt% chondroitin sulfate (CS) at room temperature, and then different concentrations of hyaluronic acid (HA), type I collagen (COL I, Collagen I), or β-tricalcium phosphate (β-TCP) are added. During thawing, the* n*-butanol is evacuated, leaving micropores inside the scaffolds. The scaffolds are then co-cultured in vitro with bone marrow mesenchymal stem cells (BMSCs) and/or chondrocytes before being transferred to the osteochondral defect area and sealed in vivo with a self-setting RSF hydrogel

In this study, we developed a pure RSF-derived composite comprising a porous scaffold and an edge sealer for effective osteochondral engineering (Scheme 1). The physicochemical, mechanical, and biological properties of the composite were optimized in vitro for the construction of a functional scaffold and self-solidifying RSF solution, which enabled not only the regeneration of cartilage and subchondral bone but also the integration between the engineered and natural tissues. Our study presents a new approach to marginal sealing around the integral *n*-butanol-inspired small aperture scaffolds with an RSF solution, laying a theoretical foundation for the clinical translation of osteochondral regeneration in the future.

## Methods and materials

### Preparation of the *n*-butanol-inspired porous scaffolds

*N*-butanol was added to a 7 wt% RSF solution at a volume ratio of 1:4 while stirring, cast into a mold, and frozen at − 20 °C for 24 h to fabricate porous RSF scaffolds. We prepared a 14 wt% RSF, 2.1 wt% COL I, 3.15 wt% CS, and 3.15 wt% HA solution. Subsequently, the CS and HA or COL I solutions were added to the RSF solution to obtain RSF/CS/HA and RSF/CS/COL I mixture solutions with mass ratios of 100:1.5:1, 100:1.5:5, 100:1.5:10, and 100:1.5:15. β-TCP solution was added to the RSF solution at a mass ratio of 1:3, 1:1, or 3:1 to obtain a mixture. Then, the solutions of RSF/CS/HA, RSF/CS/COL I, and RSF/β-TCP underwent the same procedure as described in the RSF scaffold preparation. Finally, the scaffolds were prepared.

### Morphological observations

The cross-sectional morphology of the ECM-inspired porous scaffolds was observed using a Cryo field emission scanning electron microscope (Cryo-FESEM, Zeiss Gemini SEM500) with an EDS spectrometer. After lyophilization, the ECM-inspired porous scaffolds were fractured in liquid nitrogen and sputter-coated with gold prior to observation. The diameters of the pores of the scaffolds were measured using Image J; 50 pores were randomly selected for statistical analysis.

### Porosity measurements

The porosity of the *n*-butanol-inspired scaffolds was calculated using a liquid displacement strategy with hexane. Hexane neither swelled nor contracted the porous scaffold, making it the ideal solvent. Lyophilized porous scaffolds were initially immersed in hexane, known as volume (V1), and then completely immersed with hexane through evacuation–depressurization cycles, recorded as volume (V2). The final volume was then recorded (V3) following the hexane-immersed scaffold movement. Thus, the porosity could be analyzed using the formula. $Porosity(\%)=[(V1-V3)/(V2-V3)]\times 100\%$.

### Rheological tests of a 14 wt% RSF solution

Rheological characteristics of a 14 wt% RSF solution were carried out on a Physica MCR 301 rheometer (Anton Paar GmbH, Australia) with a 25 mm parallel-plate configuration and a 1 mm gap distance. A dynamic frequency sweep experiment was performed in the range of 0.1–500 rad/s with 1% strain at 37 °C.

### Degradation and cell Fluorescence tracer in vivo

The Rhodamine B solution was added to the RSF solution to obtain a final mixture with a Rhodamine B concentration of approximately 5 ppm. Degradation experiments were conducted under accelerated degradation conditions using rats. At specified time points (three and four months), three disks were removed from the subcutaneous tissues and fixed with a 4 wt% polyformaldehyde solution containing 30 wt% sucrose for 48 h. Frozen sections were made and stained with DAPI, and the fluorescence area of Rhodamine was measured and analyzed. Chondrocytes and BMSCs were transfected with DIL (red) and DIO (green) (Beyotime, Shanghai, China) fluorescent probes, respectively. The cell labeling rate was detected by flow cytometry, and the cell scaffold complex was removed from the subcutaneous tissue on the back of nude mice after seven days, and then fixed in 4% paraformaldehyde containing 30% sucrose for 48 h. Frozen Sects. (5–8 μm) were made and DAPI staining was performed. The fluorescence areas of DIL and DIO were analyzed.

The detailed experimental method is shown in the Supplementary Information of the in vivo osteochondral repair and evaluation, including histological evaluation of cartilage repair, RNA extraction, and mRNA-seq.

### Mechanical tests

The wet scaffolds were cut into cylindrical pieces (diameter of 12 mm; thickness of about 5 mm) and balanced in deionized water for more than 24 h to completely equilibrate under wet conditions and then wiped carefully with filter paper for the removal of surficial water before the test. The mechanical properties of the scaffolds were measured with compression mode on an Instron 5565 (Norwood, MA, USA) at room temperature. The actual height of each sample was measured using the machine at a 500 N tare load, and the crosshead speed was 5 mm/min until the displacement of 50% strain. The stress–strain curves were plotted, and the compressive strength and modulus were calculated using previously reported methods. Five samples were measured for each group.

### Cell seeding and proliferation on *n*-butanol-inspired scaffolds

The rabbit knee chondrocytes and bone mesenchymal stem cells (BMSCs) were seeded with an initial density on each scaffold in 6- or 24-well cell culture plates containing 50 μL of Dulbecco’s modified Eagle’s medium (BI, USA) or α-MEM (BI, USA) supplemented with 10% fetal bovine serum, 100 U/mL penicillin, and 100 μg/mL streptomycin. The cell-seeded scaffolds were cultivated in an incubator at 37 °C and 5% CO2 for approximately 20 min, and then a 2 mL of culture medium was added to each well. The cell culture medium was replaced every 2 days. Live/dead staining was performed, and cell cytotoxicity in chondrocytes and BMSCs cocultured on the hydrogels or scaffolds (5–7 × 10^5^ cells/mL) was observed after 7 days. Live cells were stained green by intracellular esterase-catalyzed hydrolysis of Calcein AM, and dead cells were stained red by ethidium homodimer-1, which penetrated damaged membranes and bound to nucleic acids. The cell proliferation of the chondrocytes and BMSCs co-cultured in different scaffolds (1–3 × 10^6^ cells/mL) was investigated with CCK-8 assay. After 1, 3, 5, and 7 days, CCK-8 reagents were added for 2 h. Absorbance was measured at 490 nm with a microplate reader. All tests were repeated three times.

### Biocompatibility of the hydrogel

The injectability and biocompatibility of the pure RSF hydrogel were first evaluated macroscopically. A pure RSF solution (14 wt%) and Matrigel (Corning, USA) were mixed, and the mixture was stirred evenly in a mass ratio of 1:1. Meanwhile, chondrocytes (1 × 10^5^ cells/mL) were seeded into the mixed gelatin, added to a culture dish, and gelated for 3 min at 37 °C. The biocompatibility of the hydrogel was then tested. The cell membrane fluorescent probe DIL (Intrivogen, USA) was transfected into chondrocytes, images were obtained after 1 and 2 weeks, and the number and morphology of the fluorescently labeled cells were determined.

### In vivo osteochondral repair and evaluation

Surgical procedure and scaffold implantation: all animal experiments were conducted in agreement with the national legislation on the protection of animals and the National Institutes of Health (NIH) Guidelines for the Care and Use of Laboratory Animals (NIH Publication 85–23, Rev 1985) and were approved by the ethics committee of Shanghai Stomatological Hospital & School of Stomatology, Fudan University. In the defect preparation and treatment surgery, six skeletally mature and healthy male New Zealand white rabbits (age of 5 months; body weight of 3 ± 0.2 kg) were fed with a standard diet. The rabbits were subjected to 8 h of fasting and then sedated with intramuscular injection of 2 mg of xylazine (Sangon Biotech, Shanghai, China), 30 mg of ketamine (Ketanest S, Germany), and 1 mg of atropine (MedChemExpress, Shanghai, China). Anesthesia was administered after endotracheal intubation with intravenous administration of 20 mL of 2% propofol (MedChemExpress, Shanghai, China) and further maintained with inhalation of 1.5% isoflurane (Sigma, Shanghai, China), and then propofol (6–20 mg/kg BW/h) was intravenously injected. A standardized bilateral approach was managed with scheduled treatments in the hind legs with right and left knees alternating within different groups. Standardized circular chondral defects were created on the upper lateral trochlea facets with a round 5 mm dental punch. The full-thickness chondral defect with a vertical defect edge was achieved with meticulous debridement of the entire cartilage layers, including the calcified cartilage, downward to the subchondral bone plate. Particular attention was paid to ensuring that the introduced holes were perpendicular to the local subchondral bone plate. Sterile porous scaffolds containing chondrocytes or BMSCs or both (5–7 × 10^5^ cells/mL) were loaded into all defects. According to the grouping, RSF solution was injected into the gap between the spongy scaffold and the defect area with a syringe. The RSF solution is with the current user.

In situ gelation and arresting of bleeding occurred after a 3–4 min, and the joints were closed in layers. Immediate full-weight bearing was performed after surgery. A fentanyl pain patch was used only within the first 3 days postoperatively. Animals were euthanized at 4 and 4.5 months postoperatively, and the entire defect area was photographed for macroscopic evaluation. Osteochondral specimens containing the cartilage defects were retained in 4 wt% formaldehyde for 24 h and then stored in 70% ethanol. Micro-computed tomography (Micro-CT) analysis was performed, and the samples were decalcified with 5% formic acid and trimmed for histological, immunohistological, and histomorphometric analyses.

### Macroscopic evaluation of osteochondral defect repair

Two blinded experienced investigators evaluated the photographs for each group with International Cartilage Repair Society Scale (ICRS) scores.

### Micro-CT analysis

The rabbits (*n* = 6 in each group) were euthanized 4 and 4.5 months after implantation, and the femoral condyle was explanted and fixed in 4 wt% buffered paraformaldehyde. The new subchondral bone formed in the osteochondral defect area was detected with a micro-CT system (SkyScan 1176, Bruker, Belgium). The scanning parameters used were as follows: 18 μm resolution, 0.2 mm aluminum filter, 80 kV voltage, and 112 μA current. 3D reconstructions were made using mimics software provided by the company (CTVol). The lower and upper gray thresholds were set at 85 and 255, respectively. The ratio of bone volume to tissue volume was quantitatively determined.

### Histological evaluation of cartilage repair

Masson-Goldner (Masson-G), Safranin Orange/Fast Green (Saf-O/Fast Green), hematoxylin–eosin (HE) and collagen type II (Collagen II, COLII) immunohistochemical staining (IHC) were performed on paraffin-embedded tissue sects. (5–8 μm). The stains were evaluated by ImageJ.

### RNA extraction and mRNA-seq

New tissues formed in the defect areas at the 4.5th month were used in mRNA-seq, and at least three samples were collected from each group. Total RNA was extracted from freshly isolated tissues with TRIzol (Invitrogen, USA) according to the manufacturer’s instructions. Then, the total RNA was quantitatively determined by a NanoDrop and Agilent 2100 bioanalyzer (Thermo Fisher Scientific, USA). The final RNA samples were sequenced using a BGISEQ-500 platform (Shenzhen, China) and compared with the cDNA library. Low-quality tags were deleted before data analysis. Clean tags were then mapped to reference genomes and other RNA databases. The expression levels of mRNA samples were calculated with a unique molecular identifier for the evaluation of the absolute number of molecules.

### Statistical analysis

Results were expressed as the mean ± standard deviation (SD). One-way ANOVA with Tukey's post hoc test, Wilcoxon signed-rank test, and Mann–Whitney U test was used where appropriate. Calculations were performed using GraphPad Prism version 8.0.2 (GraphPad Software, Inc., San Diego, USA). The level of significance was set as follows: * represents *p* < *0.05*, ** represents *p* < *0.01*, *** represents *p* < *0.001* and **** represents* p* < *0.0001.*

## Results

### Preparation and characterization of *n*-butanol-inspired small pore size scaffolds and RSF solution

**Fig. 1:**
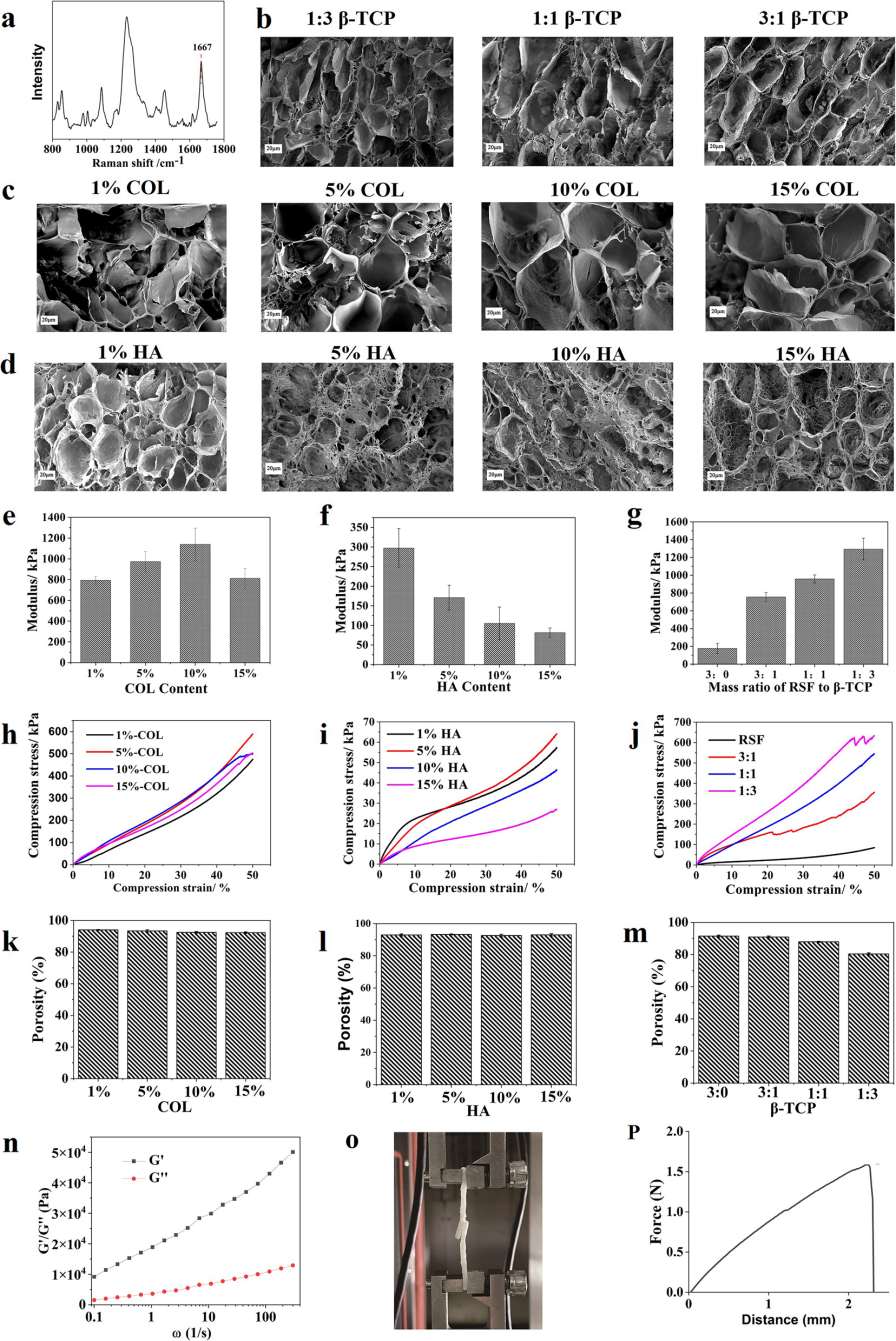
Preparation and characterization of scaffolds with small pore sizes inspired by *n-*butanol and RSF solution: **a** Raman spectrum of RSF scaffold; **b–d** Scanning electron microscopy (SEM) images of the 3D spatial structure of the scaffold; **e–g** Compression modulus of different RSF scaffolds; **h–j** Compression stress–strain curves; **k–m** Porosity of the scaffolds; **n** Frequency sweep of 14 wt% RSF solution with 1% strain; **o** Tensile test of a 14 wt% RSF solution; **p** Displacement extension diagram of a 14 wt% RSF solution

Successful preparation of RSF scaffolds through an innovative combination of *n-*butanol addition induces the conformational transition of RSF from a random and/or helical structure to β-sheets [19]. Figure 1a shows the Raman spectrum of the RSF scaffolds. It clearly shows that the amide I band is located at 1667 cm^−1^, which is typical for a β-sheet structure. Scheme 1 shows a representation of the fabrication process for obtaining 3D porous RSF scaffolds.

The aperture of the ECM-added scaffolds varies slightly according to the mass ratio of RSF, HA, COL I, and β-TCP. Figure 1b–d reveal that the surface of the RSF scaffold is rough; the pore size of the 5% HA group is 41.5 ± 8.8 μm, the 10% COL group is 70.6 ± 15.5 μm, and the 1:1 β-TCP group is 20.5 ± 9.5 μm (Table 1). The compression modulus of the 10% COL and 1:3 β-TCP groups is the highest among the other groups, reaching up to 1100 kPa (Fig. 1 e and g). That of the 1:1 β-TCP group is also high at 900 kPa (Fig. 1g), but that of the 5% HA group is much lower, at only 175 kPa (Fig. 1f). When the compressive strain is 20%, the compressive stress of the 10% COL and 1:1 β-TCP groups is the same at 200 kPa (Fig. 1h–j), whereas that of the 5% HA group is as low as 30 kPa. Despite this result, the porosity of almost all groups is approximately 90% (Fig. 1k–m).

**Table 1:** Pore size of different RSF scaffolds

**Name**	**1% HA**	**5% HA**	**10% HA**	**15% HA**
Pore size (μm)	25.5±8.4	41.5±8.8	21.5±8.3	40.4±15.3
**Name**	**1% COL**	**5% COL**	**10% COL**	**15% COL**
Pore size (μm)	28.9±12.7	41.8±17.6	70.6±15.5	33.8±16.7
**Name**	**3:1 β-TCP**	**1:1 β-TCP**	**1:3 β-TCP**	
Pore size (μm)	74.0±27.6	20.5±9.5	14.9±6.8	

At room temperature, a 14 wt% RSF solution is in a solution state, which is sensitive to external forces. However, it immediately turns into a gel state upon encountering shear and frictional forces. Figure 1n illustrates the rheological behavior of the 14 wt% RSF solution used as a glue in this study. Even under extremely low shear force (ω = 0.1), the storage modulus G’ is larger than loss modulus G”, indicating the formation of an RSF hydrogel. Figure 1o and p demonstrate the adhesion of two pieces of RSF scaffolds after 14 wt% RSF solution was used, showing excellent bonding performance.

The results in Fig. 1 show that the aperture of the 10% collagen group (10% COL, approximately 70 μm) is larger than that of the HA groups (40 μm), which is convenient for the transportation of nutrients in the stent. At the same time, the 10% COL group has the highest compression modulus (1100 kPa) for resisting external pressure, which is probably the ideal option for the middle layer of cartilage. At the same time, the 10% COL group has the highest compression modulus (1100 kPa) for resisting external pressure, which is likely the ideal option for the middle layer of cartilage. A 14 wt% RSF solution is also used as an adhesive to bond the porous scaffold and the tissue around the defect area.

### Effects of in vitro chondrogenesis for *n-*butanol-inspired small aperture scaffolds

#### Screening ingredients in vitro at different concentrations for n-butanol-inspired small aperture scaffolds

GAGs and COL II are the main ECM components of knee cartilage; thus, the contents of double-stranded DNA (dsDNA), total GAGs, and collagen were detected separately after cells were co-cultured with scaffolds for seven days in vitro. Compared with the other groups, 10% COL, 1:1 β-TCP groups had the highest production of dsDNA, total GAG, and total collagen. The 5% HA group had relatively high dsDNA and total GAG content, but a slight decreased total collagen content (Fig. S1b–j, Supporting Information). Natural cartilage was roughly divided into two layers: cartilage and subchondral bone, in which 5% HA and 10% COL may be ideal for a cartilage layer (Fig. S1a, Supporting Information).

**Fig. 2:**
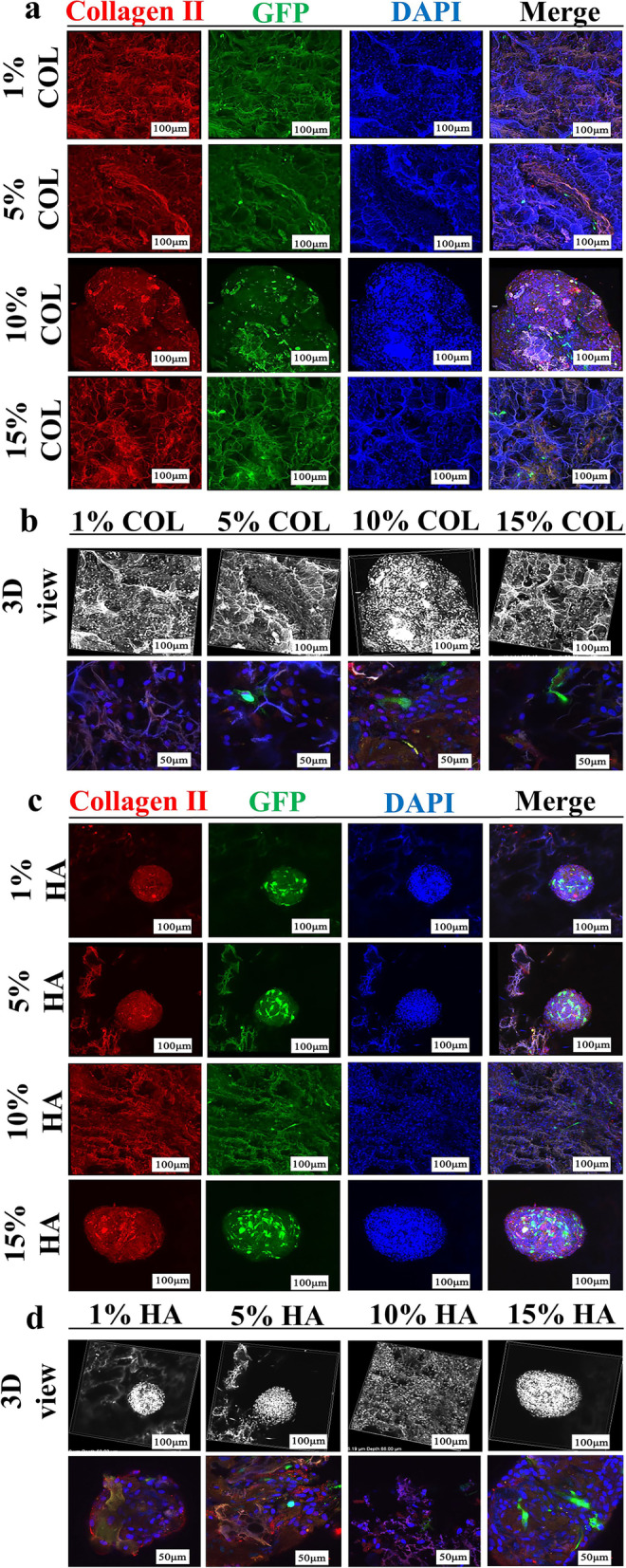
Screening ingredients at different HA/COL/β-TCP concentrations for *n-*butanol-inspired small aperture scaffolds in vitro: **a–d** type II collagen immunofluorescence staining of chondrocytes on RSF/HA and RSF/Collagen I materials after seven days of culture

The COL II protein is a commonly used marker to assess the presence and quality of cartilage in tissues, including knee cartilage. The use of COL II immunofluorescence staining allows researchers to visualize the expression of this protein within the tissue, providing insight into the state of the cartilage. In the experiment, chondrocytes and BMSCs were seeded onto the scaffolds and allowed to grow for one week. The cells were stained with COL II immunofluorescence staining after that certain period of time. The 10% COL group showed the highest number of GFP-labeled chondrocytes and production of COL II (red spots). In addition, the different collagen groups were covered with a large number of nuclei from 3D views and magnification data (Fig. 2a–b). The number of cells in the HA group is similar to that in the collagen group (Fig. 2c–d). However, the mixed cells aggregated spherically on the HA scaffolds, probably due to the high viscosity of HA itself. When more HA is added, the formability becomes unacceptable [20]. This finding was proven by the phenomenon that the 15% HA exhibited an extremely fragile feature in vitro. For porous scaffolds, the cells’ nutrient supply on the scaffolds has always attracted considerable attention [21]. To solve this issue within the cellular sphere, an HA scaffold was placed as the upper layer of the cartilage gradient structure to receive nutrition from the subcutaneous and surrounding cartilage defect areas at an early date.

**Fig. 3:**
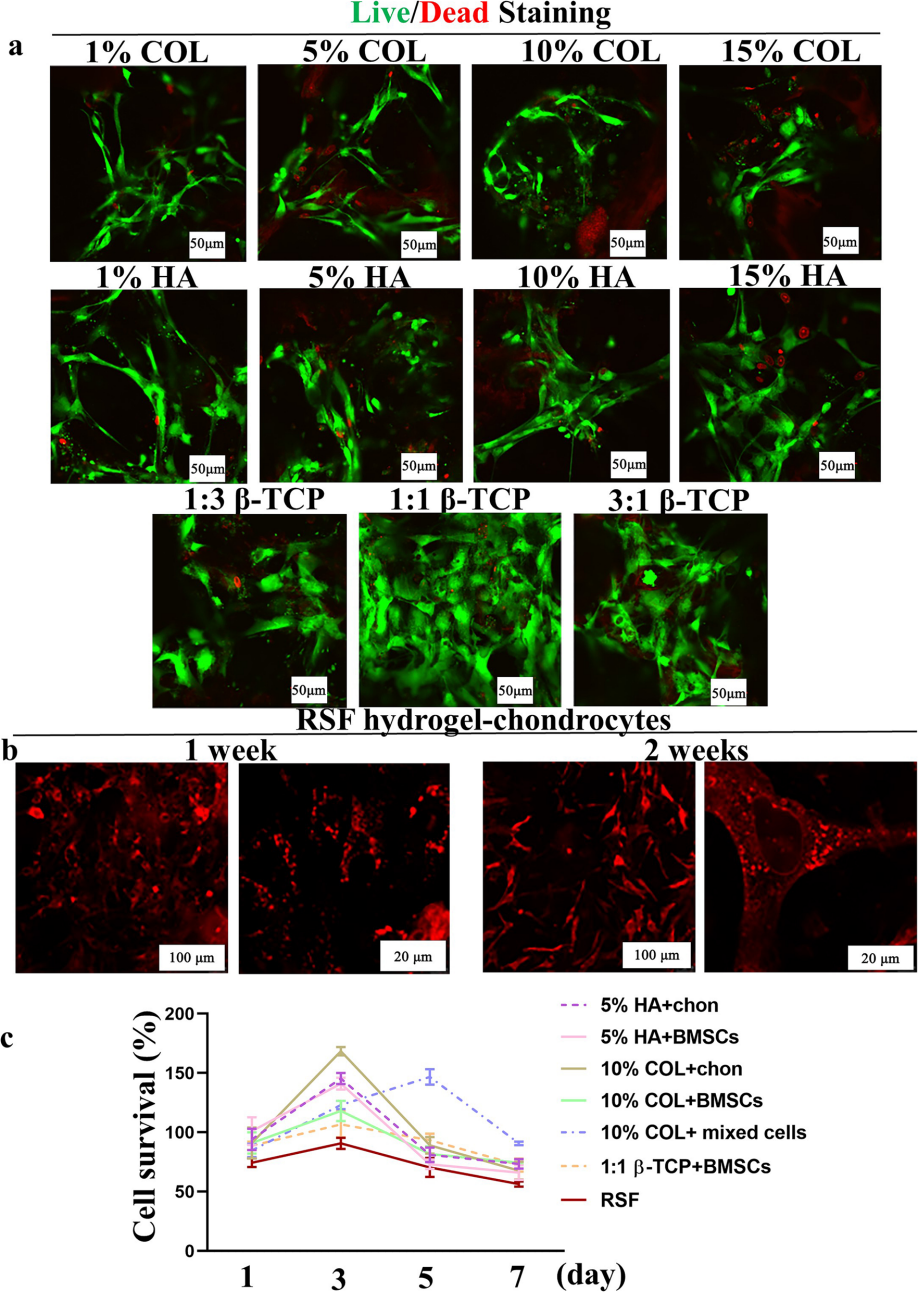
Cell growth and proliferation in the 14 wt% RSF solution and RSF scaffolds in vitro: **a** Live/dead staining of chondrocytes and/or BMSCs on materials after co-culture for seven days; **b** Cell growth state of chondrocytes on the 14 wt% RSF solution cultured in normal medium for one and two weeks; **c** Cell viability of chondrocytes and/or BMSCs on RSF/HA, RSF/COLI, and RSF/β-TCP scaffolds

For clinical applications, favorable biocompatibility is an essential requirement for scaffolds [22]. The RSF hybrid scaffolds and RSF solution were biocompatible, non-hemolytic, and supported cell adhesion, growth, and proliferation (Fig. 3). As shown in Fig. 3a, most chondrocytes and BMSCs were alive (green fluorescence) in the HA, COL, and β-TCP groups, with only a few dead cells present (red fluorescence). Furthermore, the number of live cells in the 1:1 β-TCP group was significantly higher than in other groups. Chondrocytes co-cultured in the RSF hydrogel for two weeks still stretched and grew well (Fig. 3b). Cell viability, compared with the control group (RSF group), did not decrease when the cells were co-cultured with the scaffolds for one, three, five, or seven days, as detected by the CCK-8 assay (Fig. 3c).

On the basis of these research results, we designed that Groups A, B, and C were cultured with different cells. Group D added a layer of TCP as the subchondral bone [23] based on Group C, and Group E added RSF solution as a sealing agent based on Group D. Moreover, the 10% COL middle layer was co-cultured with chondrocytes and BMSCs in a ratio of approximately 2:1 [24]. Chondrocytes created a differentiation microenvironment for BMSCs to become chondrocytes, whereas BMSCs provided various growth factors for the chondrocytes [25] (Fig. 5b).

### Effects of *n*-butanol-inspired small aperture scaffolds and/or RSF hydrogel in vivo

#### Degradation of n-butanol-inspired small aperture scaffolds in vivo

**Fig. 4:**
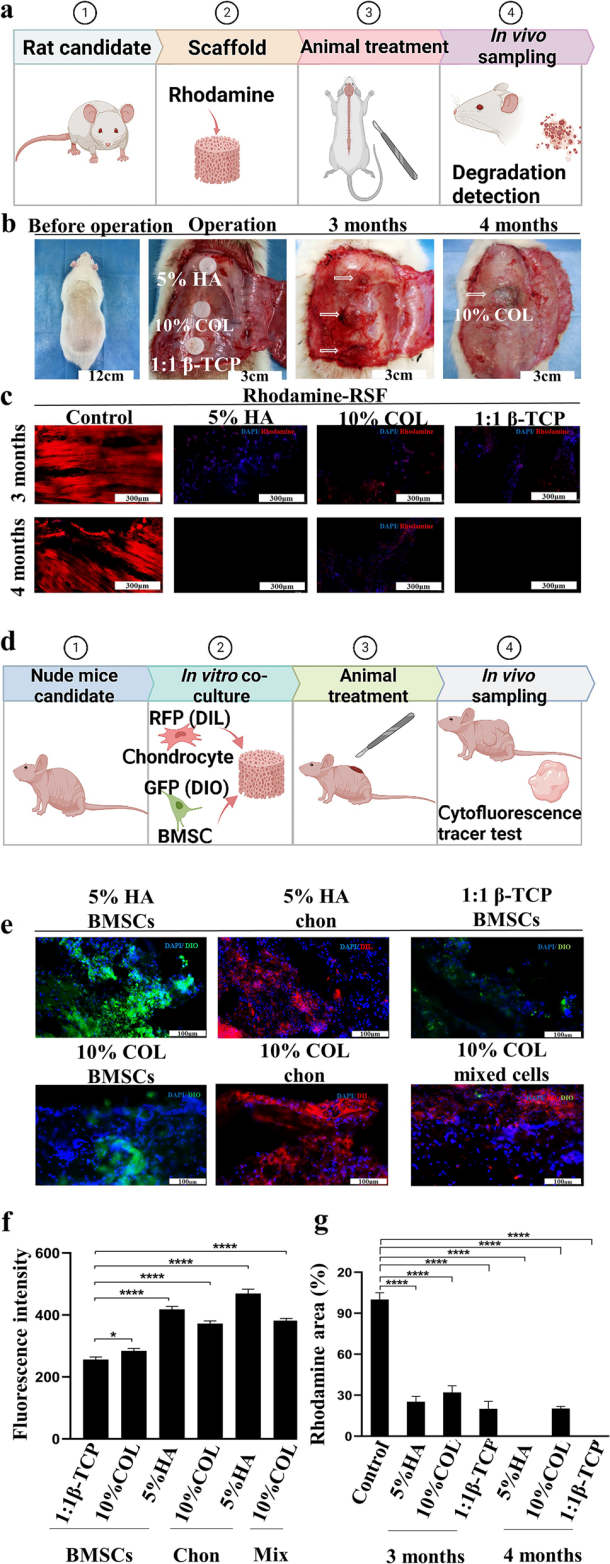
The degradation and cell fluorescence tracer effects of *n-*butanol-inspired small aperture scaffolds in vivo were assessed. **a** Surgical procedures involving RSF-rhodamine with subcutaneous transplantation in rats were conducted, **b** with immediate implantation conditions at 0 months and degradation conditions at three and four months. **c** DAPI staining was applied at three and four months to show the rhodamine area distribution. **d** A schematic procedure for cell fluorescence tracer testing in nude mice for seven days was described, **e** with the area of DIL (red)-labelled chondrocytes (chon) and DIO (green)-labelled BMSCs implanted on the materials for seven days being estimated. **f, g** ImageJ was used to quantify the DIO, DIL, and Rhodamine areas. * represents *p* < *0.05*, ** represents *p* < *0.01*, *** represents *p* < *0.001*, and **** represents *p* < *0.0001*

Almost all the scaffolds were degraded in rat subcutaneous tissue by the fourth month in the 5% HA, 10% COL, and 1:1 β-TCP groups (Fig. 4). A consensus that the degradation products of RSF are mainly amino acids that are absorbed by our body without causing rejection has been reached. Moreover, the ideal degradation time needs to match the time of nascent tissue formation. Maintaining a scaffold’s microstructure is easy, but obtaining an inflammatory response takes time [7]. Therefore, we added rhodamine to the RSF scaffolds and then implanted (1 cm in diameter, 3 mm in thickness) subcutaneously in rats, and finally collected them after three and four months (Fig. 4a). In the third month, the three groups’ scaffolds were wrapped by subcutaneous tissue, and their volumes were significantly reduced (Fig. 4b). The 5% HA, 1:1 β-TCP group degraded three-quarters of their total volume, and the 10% COL group arrived at two-thirds. At the fourth month, the scaffolds in the first two groups could not be seen. However, the remaining part in the latter group approximately accounted for one-fifth after analysis (Fig. 4c and g). On the 30th day, the in vitro mass loss rates of the RSF/HA groups 1%, 5%, 10%, and 15% were about 85%, 80%, 78%, and 72%, respectively. By contrast, the mass loss rates were approximately 85%, 80%, 75%, and 70% in the 1%, 5%, 10%, and 15% RSF/COL groups, respectively. The mass ratios of RSF to β-TCP were 3:0 and 1:3, and their mass loss rates were as high as 90% initially and then decreased to 25% (Fig. S2, Supporting Information). As the amounts of type I collagen and β-TCP increased, the degradation rate gradually slowed down. Type XIV collagenase degrades the β-fold region in the silk protein but may have no effect on type I collagen and β-TCP.

#### Cell fluorescence tracer of n-butanol-inspired small aperture scaffolds in vivo

A large number of living cells can still be seen after the scaffold loaded with rabbit cells is implanted under the skin of nude mice for one week, indicating that the environment and pore size of the scaffold is conducive to cell growth (Fig. 4d–f). Cell survival time in vivo has always been a concern because they may undergo apoptosis due to a host’s immune rejection and insufficient nutrients. Rabbit chondrocytes labelled with different fluorescence (DIL and DIO, Fig. S3, Supporting Information) were loaded onto scaffolds (Fig. 4d), and then transferred and tracked in nude mice for one week. The 5% HA group had a large number of chondrocytes, and they aggregated in clumps, consistent with the results in vitro. In addition, green and red fluorescent cells were seen in the stem cell groups and the mixed culture cell groups (Fig. 4e–f).

#### Effects of n-butanol-inspired small aperture scaffolds and RSF hydrogel on osteochondral defects in vivo

**Fig. 5:**
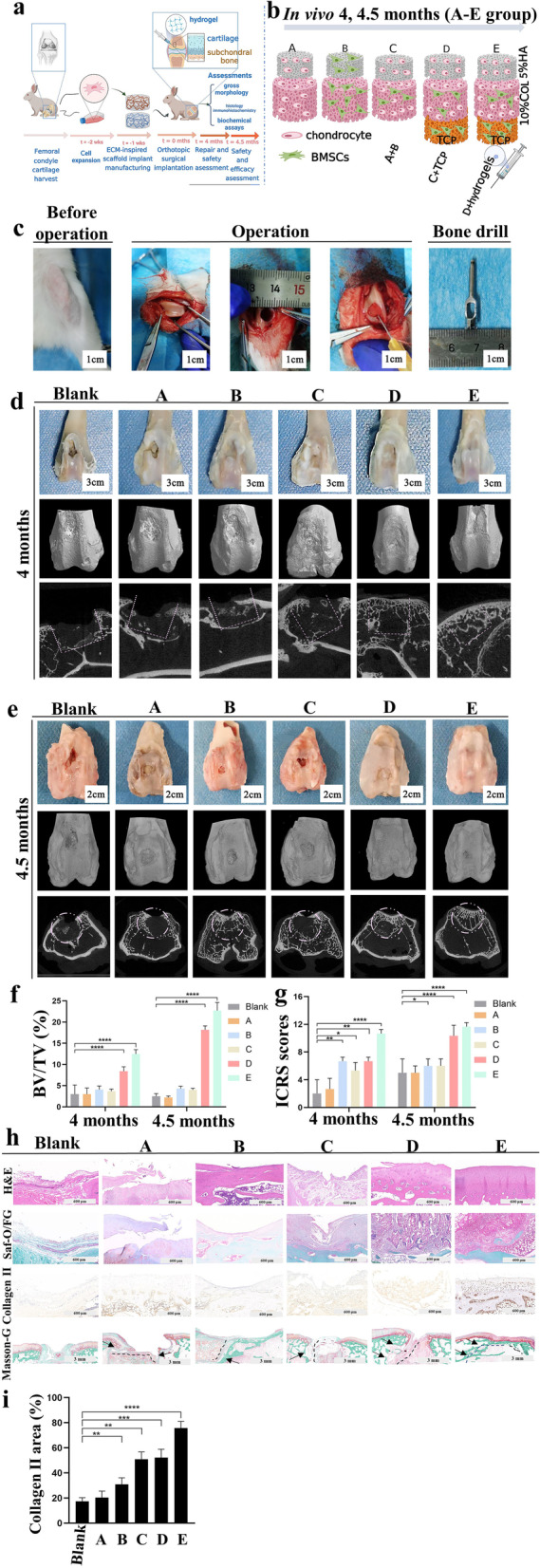
Osteochondral engineering using the all-RSF-derived composite in vivo: **a** Procedures for the hybrid scaffolds and RSF solution application; **b** Schematic of each group are as follows: Group A: All chondrocytes; Group B: All BMSCs; Group C: Mixed cells; Group D: Group C + β-TCP; Group E: Group D + 14 wt% RSF solution; **c** Surgical procedures for the use of scaffolds combined with RSF solution in the osteochondral repair; **d, e** Gross images and micro-CT images; **f** BV/TV (%) values of micro-CT bone remodeling were quantitatively measured; **g** ICRS macroscopic repair scores were quantified; **h** H&E staining, Saf-O/Fast Green staining, COL II IHC, and Masson–Goldner staining (the black arrow indicates the junction between the material and the tissue surrounding the defect) of repaired cartilage subchondral bone at four months post-surgery; **i** Relative expression analysis of COL II IHC with ImageJ

*N-*butanol-inspired scaffolds sealed by RSF hydrogels are a promising candidate for osteochondral defects, as revealed by the results in vivo (Fig. 5). New Zealand white rabbits were injected with ketamine, and a full-thickness cartilage defect (5 mm diameter, 4 mm depth) was created. Subsequent treatments consisting of six groups were assessed through observation and tissue collection for a long time at 4 and 4.5 months (Fig. 5a–c). Macroscopically (ICRS scores), the four remaining groups displayed faster cartilage formation than the A, blank groups. The D and E groups had the highest healing scores, whereas only ≈20% closure was achieved in the A groups at 4 and 4.5 months. The micro-CT data also showed that group E had more osteogenesis than the other groups (Fig. 5d–g).

Results from histological analysis by H&E, Masson-G, Saf-O/FG, and COL II IHC were consistent with the wound healing rate (Fig. 5h–i). The statistical measurement of cartilage and subchondral bone tissue showed that the superficial cartilage formation of the E group was faster than those of the other groups at a later stage of healing (4, 4.5 months). Specifically, defects from group E after 4.5 months of treatment were almost healed and exhibited a typical histological architecture similar to the rabbit knee joint, which appeared mostly scarless and had the greatest well-defined anatomy of cartilage and subchondral bone (Fig. 5h). In Masson-G, slices with a reduced field of view, implants, and surrounding tissues were merged. Thus, distinguishing the boundaries (the black arrow) of the scaffolds in the E group becomes impossible. However, cavities (the black arrow) can be seen between the materials and tissues in the other groups (Fig. 5h).

### Mechanism of *n-*butanol-inspired small aperture scaffolds and RSF hydrogel on chondrogenesis regulation in vivo and in vitro

**Fig. 6:**
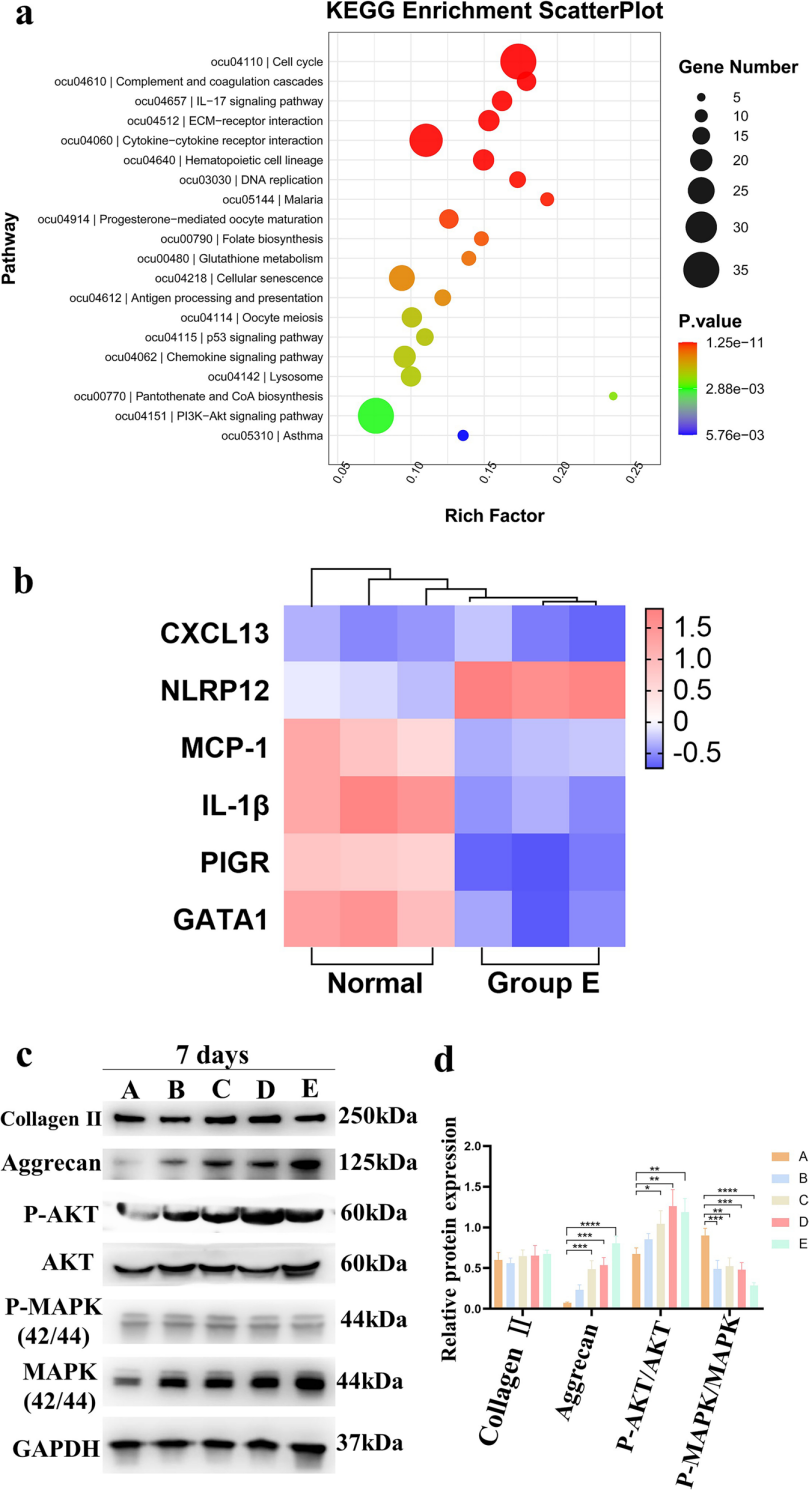
mRNA-seq and Western blot analysis: **a** Top 20 most enriched scatterplots and bar plot in KEGG analysis, with the *p-value* of 0.01 (red) and 0.05 (blue) highlighted to indicate the significance of the results based on genome background enrichment; **b** Heat map of differentially expressed genes between Group E and the control for 4.5 months in vivo; **c** Protein levels of collagen II, aggrecan, P-AKT, and P-MAPK in C28/I2 and human mesenchymal stem cells cultured with different groups were determined by Western blot after seven days in vivo; **d** Relative expression analysis of Western blot with ImageJ. * represents *p* < 0.05, ** represents *p* < 0.01, *** represents *p* < 0.001, and **** represents *p* < 0.0001

Analysis of the mRNA-sequencing (mRNA-Seq) suggested that Group E exhibited increased NLRP12 (a gene with anti-inflammatory properties), decreased IL-1β expression, and downstream immune and inflammatory responses, but did not inhibit cell proliferation and replication. Cell scaffold complexes might induce cartilage formation through the AKT and MAPK pathways (Fig. 6). mRNA-Seq was used to assess changes in messenger RNA (mRNA) levels in the chondrogenesis regulation cultured in vivo for 4.5 months in the normal and E groups. Compared with the control groups, the E group had 968 differentially expressed genes, including 340 highly expressed genes and 628 lowly expressed genes (Fig. S4, Supporting Information). Notably, KEGG pathway analysis of the differentially expressed genes associated with the chondrogenesis regulation revealed the cytokine–cytokine receptor interaction, cell cycle and proliferation, PI3K-Akt, and MAPK signaling pathways. The differentially expressed genes associated with the immune system revealed that host defense mechanisms, such as NOD-like receptor and IL-17, caused a negative regulation of the “NOD-like receptor signaling pathway” in the E group, including the signal transducer and activator of genes, such as NLRP12 and IL-1β. NLRP12 activity has been shown to decrease in response to certain infections and pro-inflammatory molecules [26]. IL-1β is a key mediator of the inflammatory response and is involved in diverse physiological and pathological processes, including inflammation, cancer, and immunity [26]. Our study also found that group E did not affect the expression of the chemokine CXCL13 in “IL-17” signaling pathways. CXCL13 is a well-known inflammatory and structural cell chemoattractant that plays a pivotal role in the migration of keratinocytes during epithelial repair and in angiogenesis [27] (Fig. 6a–b). In addition, chondrocytes (C28/I2) (Fig. S5, Supporting Information) and human mesenchymal stem cells grew on small aperture scaffolds and secreted cartilage matrix (COLII, aggrecan) possibly by activating Akt and MAPK molecules, which were detected by western blot in vitro, verifying the in vivo results (Fig. 6b–c).

## Discussion

The most important factor for the success of tissue engineering is understanding the complexity of the biological system. Researchers need to optimize the structure and scaffold materials and ensure that the cells on the scaffold can survive for as long as possible. The integration of the scaffold and the host tissue is also important to integrate the implanted complex into the host tissue and achieve the desired effect.

Cartilage-to-cartilage integration is exceedingly difficult to achieve given that the cartilage is a highly specialized tissue that requires particular conditions for integration. Lateral integration remains a significant issue because the safety and efficacy of these measures have yet to be determined [28]. Marginal sealing hydrogel based on high-concentration (14 wt%) RSF solution possesses sufficient viscosity to stabilize sponge scaffolds with initial stability and biomechanical buffer; it fills small to medium sized gaps and fractures, creating a tight seal that prevents the leakage of fluids and gases. Generally, changes in pH or temperature or the presence of shear force trigger the formation of a hydrogel and a β-sheet from a high-concentration RSF solution [29]. The formation of the hydrogel and β-sheet is advantageous for RSF solutions because it increases their mechanical strength, flexibility, and durability. These properties make RSF solutions more suitable for use in medical applications, such as wound healing and tissue engineering [30]. The results of the rabbits in vivo indicate that the production of COL II and the degree of wound healing in Group E were the best among all the groups. After four months, gaps (indicated by black arrows) were found between the materials and the surrounding tissues in Groups A, B, C, and D, as shown in Masson-G (Fig. 5h). However, the edge of the material in Group E was difficult to distinguish. Hydrogen bonds, hydrophobic interactions, and electrostatic interactions among the native tissue surface, RSF scaffold, and RSF hydrogel may provide strong adhesion [31]. In addition, the degradation time and product of the scaffold are key factors for the success of cartilage repair. In vivo results at 4 and 4.5 months showed that RSF materials have controllable degradability and excellent biocompatibility. The overall trends of the degradation results of 5% HA and 10% COL groups were consistent in vivo and in vitro but inconsistent in the 1:1β-TCP group. β-TCP was easily absorbed by subcutaneous tissues in vivo, and type XIV collagenase had no activity in vitro. After the Masson-G staining in rabbits for 4 months, the RSF/COL scaffold in the middle layer was not completely degraded, and the hole outline inside the scaffold was obvious, but the edge of the scaffold was blurred. The RSF/HA scaffold on the surface of the cartilage and the RSF/β-TCP scaffold of the subchondral bone disappeared. This result was consistent with subcutaneous degradation results in rats. No serious inflammatory reaction of the degradation products was detected by mRNA-Seq and HE staining, although the 10% COL group did not completely disintegrate, providing enough time for surrounding tissues to climb into the scaffold.

The microstructure of the scaffold material in this study is discussed. *N*-butanol is innovatively used as a self-emulsifier to form an oil-in water emulsion, and the RSF small-aperture scaffold is prepared by freezing and thawing such an emulsion. The SEM images show that the scaffold is composed of a mesh-like structure made up of interconnected macropores, with pore size ranging from 10 μm to 100 μm. The pore size of the scaffold can be easily adjusted by changing the concentrations of the foaming agent and the freezing and thawing temperatures. The optimal pore size for chondrocyte growth is still controversial. This study confirmed that the *n-*butanol-driven and BMSC-mixed scaffolds have a good chondrogenic induction ability in vitro and in vivo, but the pore size of the 10%COL layer is approximately 70 μm, and the 5%HA and 1:1β-TCP layer is approximately 40 μm, which is inconsistent with 200–500 μm in numerous previous types of research [32]. Moreover, faster migration occurs in scaffolds with a pore size of 12 μm (equivalent to cell size) than in those with pore sizes of 7 μm (smaller than cell size) and 17 μm (larger than cell size) [8]. The defects treated with an interconnected porous structure of 300–500 ​μm 3D-printed scaffolds seeded with chondrocyte-like cells exhibited significantly increased cartilage formation for up to 15 weeks compared with empty defects [33]. The small aperture scaffold has a tighter knit structure, which limits the diffusion of oxygen through the scaffold, resulting in lower oxygen tension [34]. The combination of small pore sized scaffolds and hypoxia can create a specialized environment for chondrocytes. The chondrocytes may be able to differentiate and signal more effectively in a more natural environment, where oxygen level is low. Hypoxia can also be used to regulate the number of growth factors, such as VEGF, and induce cell survival, proliferation, and differentiation [35]. However, the optimal pore size varies depending on the type of chondrocyte cells studied and the type of scaffold used. The specific requirements should be considered when selecting the optimal pore size for chondrocyte growth.

In summary, this study presents three novel points. *N*-butanol is combined with freeze and thaw to create 3D porous scaffolds, and the temperature and proportion of *n*-butanol can be adjusted to change the size of the scaffolds’ pores for cartilage regeneration. The optimal pore size for cartilage growth is still debated. However, this study discovered that cartilage can grow, reproduce, and secrete type II collagen on a very small pore size scaffold, different from the large pore size scaffolds used by many researchers in the past. This finding may be related to the survival of natural cartilage in hypoxic environments. The horizontal integration of host tissue and scaffold is the third point. A high-concentration RSF solution (14 wt%) without any other chemical additives is transformed into an RSF hydrogel when subjected to compression, tensile, and shear forces due to β-sheet transformation. The knee joint is a sports joint, which has been used effectively in this study. RSF hydrogel may provide a medium for nutrient exchange and signal transmission for cells implanted from the outside and cells in vivo.

## Conclusion

A promising hybrid scaffold based on RSF with intrinsic chondrogenesis properties can be developed as a new implant to promote cartilage defect healing. After defect embedding, the scaffold is strengthened in situ by an injectable RSF solution. As a result, the scaffolds display repairability and ideal initial stability, which is important for preventing local stress damage. Notably, in vitro, the hybrid scaffold stimulates the generation of cartilage-related markers without the exogenous addition of costly biological factors. In vivo studies reveal that the scaffold with a 14 wt% RSF solution accelerates cartilage production with clear layers. In addition to demonstrating chondrogenesis functions, the implant is shown to have excellent biocompatibility at 4.5 months. In summary, the *n-*butanol-inspired small aperture scaffolds with edge-sealed hydrogel are a promising new implant material for the management of osteochondral defects.

## Supplementary Information

**Additional file 1:** **Fig. S1.** Screening ingredients at different concentrations for *n*-butanol-inspired small aperture scaffolds *in vitro*: a) Illustration of cell-scaffold complex preparation; b-j) dsDNA, total GAG, and total collagen content of different HA, COLI, and β-TCP mass ratios of the small aperture scaffolds after co-culturing with chondrocytes or BMSCs for seven days. * represents *p < 0.05*, ** represents *p < 0.01*, *** represents *p < 0.001 *and **** represents *p < 0.0001.* **Fig. S2.** The mass loss rate of RSF porous scaffolds *in vitro* under the action of type XIV collagenase at 37 ℃. a) It shows the RSF/HA group, and b) the RSF/COL group. c) Groups with RSF and β-TCP mass ratios of 1:3, 1:1 and 3:1. **Fig. S3.** Labelling rate detection of cell fluorescent probes by flow cytometry. a) 99.84% labelling rate of chondrocytes with a red fluorescent probe. b) 84.32% labelling rate of BMSCs with a green fluorescent probe. **Fig. S4.** mRNA-seq analysis. a) The overall distribution of differentially expressed genes in osteochondral samples from the control and E groups after 4.5 months is shown. Red represents significantly up-regulated differentially expressed genes, blue represents significantly down-regulated differentially expressed genes, and grey dots represent non-significantly differentially expressed genes. b) Compared to the control groups, the E groups have 968 differentially expressed genes, including 340 highly expressed genes and 628 low-expressed genes. **Fig. S5.** Cell identification. a) Immunofluorescencestaining of collagen type II, aggrecan, and SOX9 of the human chondrocyte line C28/I2. b) IF staining of collagen type II and aggrecan of rabbit chondrocytes from the knee joint.

## Data Availability

The datasets used and/or analysed during the current study are available from the corresponding author upon reasonable request.

## Abbreviations

RSF: Regenerated silk fibroin
BMSCs: Bone mesenchymal stem cells
Chon: Chondrocytes
GAGs: Glycosaminoglycans
Collagen I, COL I: Type I Collagen
Collagen II, COL II: Type II Collagen
HA: Hyaluronic acid
ECM: Extracellular matrix
CS: Chondroitin sulfate
SEM: Scanning electron microscope
CCK-8: Cell Counting Kit-8
H&E: Hematoxylin & eosin
Saf-O/FG staining: Saf-O/Fast Green staining
Masson-G staining: Masson–Goldner staining
IHC: Immunohistochemical staining
