# Supplementary material for: Regenerated silk fibroin based on small aperture scaffolds and marginal sealing hydrogel for osteochondral defect repair
Source: Biomater Res. 2023 May 19;27:50. doi: 10.1186/s40824-023-00370-1 (PMC10197849; doi:10.1186/s40824-023-00370-1)
Supplement: Supplementary file 1 — Additional file 1: Fig. S1. Screening ingredients at different concentrations for n-butanol-inspired small aperture scaffolds in vitro: a) Illustration of cell-scaffold complex preparation; b-j) dsDNA, total GAG, and total collagen content of different HA, COLI, and β-TCP mass ratios of the small aperture scaffolds after co-culturing with chondrocytes or BMSCs for seven days. * represents p < 0.05, ** represents p < 0.01, *** represents p < 0.001 and **** represents p < 0.0001. Fig. S2. The mass loss rate of RSF porous scaffolds in vitro under the action of type XIV collagenase at 37 ℃. a) It shows the RSF/HA group, and b) the RSF/COL group. c) Groups with RSF and β-TCP mass ratios of 1:3, 1:1 and 3:1. Fig. S3. Labelling rate detection of cell fluorescent probes by flow cytometry. a) 99.84% labelling rate of chondrocytes with a red fluorescent probe. b) 84.32% labelling rate of BMSCs with a green fluorescent probe. Fig. S4. mRNA-seq analysis. a) The overall distribution of differentially expressed genes in osteochondral samples from the control and E groups after 4.5 months is shown. Red represents significantly up-regulated differentially expressed genes, blue represents significantly down-regulated differentially expressed genes, and grey dots represent non-significantly differentially expressed genes. b) Compared to the control groups, the E groups have 968 differentially expressed genes, including 340 highly expressed genes and 628 low-expressed genes. Fig. S5. Cell identification. a) Immunofluorescencestaining of collagen type II, aggrecan, and SOX9 of the human chondrocyte line C28/I2. b) IF staining of collagen type II and aggrecan of rabbit chondrocytes from the knee joint. [file 40824_2023_370_MOESM1_ESM.docx]

**Supporting Information**

Regenerated silk fibroin based on small aperture scaffolds and marginal sealing hydrogel for [osteochondral defect repair](https://pubmed.ncbi.nlm.nih.gov/35322596)

Yinyue Luo^1,2^, Menglin Xiao^1,3^, Bushra sufyan Almaqrami^4^, Hong Kang^5^, Zhengzhong Shao^1,3^, Xin Chen^1,3*^, Ying Zhang^1,2*^

1. Department of Preventive Dentistry, Shanghai Stomatological Hospital & School of Stomatology, Department of Macromolecular Science, Fudan University, Shanghai, 200001 China;

2. Shanghai Key Laboratory of Craniomaxillofacial Development and Diseases, Fudan University, Shanghai, 200002 China;

3. State Key Laboratory of Molecular Engineering of Polymers, Laboratory of Advanced Materials, Fudan University, Shanghai, 200433 China;

4. Department of Orthodontics, Ningbo Dental Hospital, Ningbo, Zhejiang, 315032 China.

5. Department of Temporomandibular joint and Occlusion, School/Hospital of Stomatology, Lanzhou University, Lanzhou, Gansu, 730013 China.

Yinyue Luo and Menglin Xiao contributed equally to this work.

*Corresponding author: Professor Chen Xin and Professor Zhang Ying are co-corresponding authors.

Electronic address: [chenx@fudan.edu.cn](mailto:chenx@fudan.edu.cn), zhangyingcmu@vip.163.com/sophiazhang@163.com

1. **Supporting figures**

| 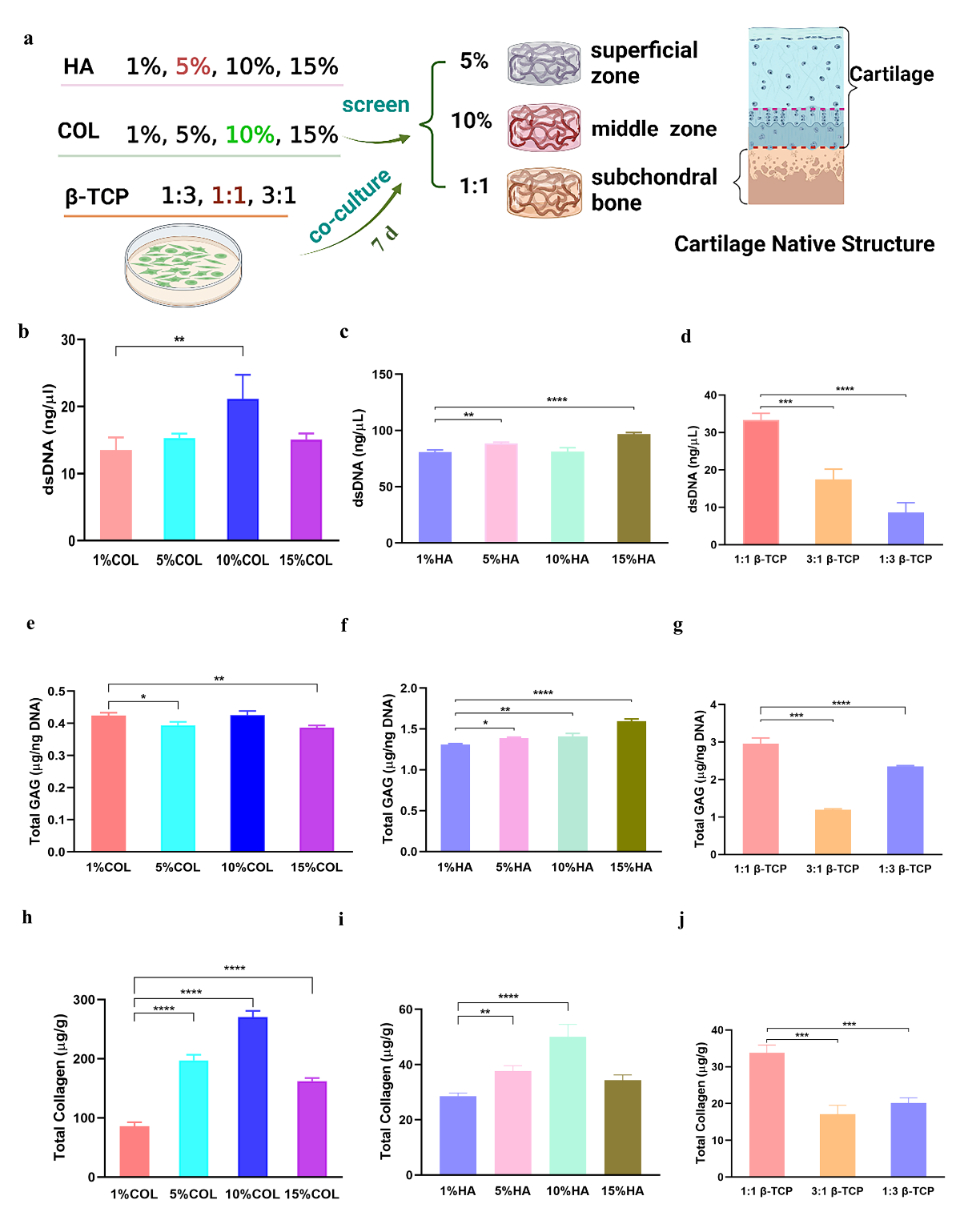 |
| --- |
| **Fig. S1**  Screening ingredients at different concentrations for n-butanol-inspired small aperture scaffolds *in vitro*: a) Illustration of cell-scaffold complex preparation; b-j) dsDNA, total GAG, and total collagen content of different HA, COLⅠ, and β-TCP mass ratios of the small aperture scaffolds after co-culturing with chondrocytes or BMSCs for seven days. * represents *p < 0.05*, ** represents *p < 0.01*, *** represents *p < 0.001* and **** represents *p < 0.0001.* |

| 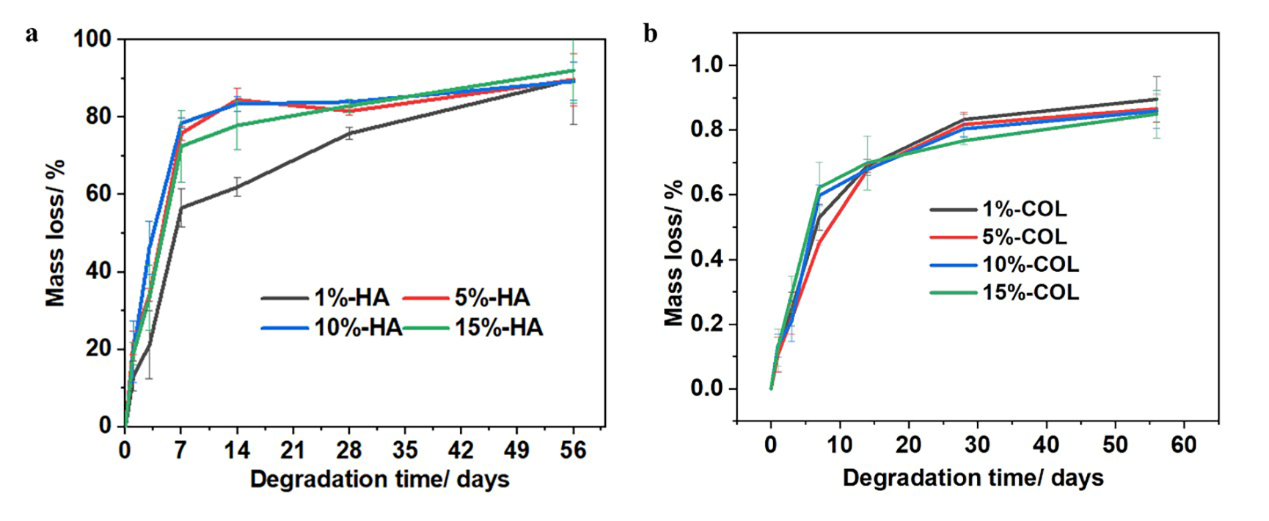 |
| --- |
| 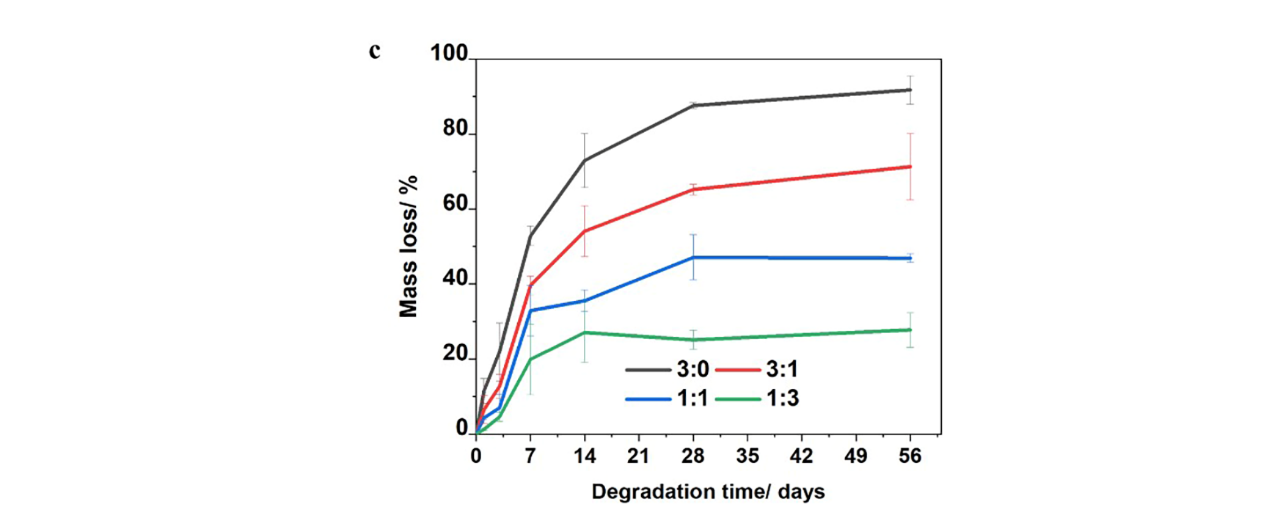 |
| **Fig. S2**  The mass loss rate of RSF porous scaffolds *in vitro* under the action of type XIV collagenase at 37 ℃. a) It shows the RSF/HA group, and b) the RSF/COL group. c) Groups with RSF and β-TCP mass ratios of 1:3, 1:1 and 3:1. |

| 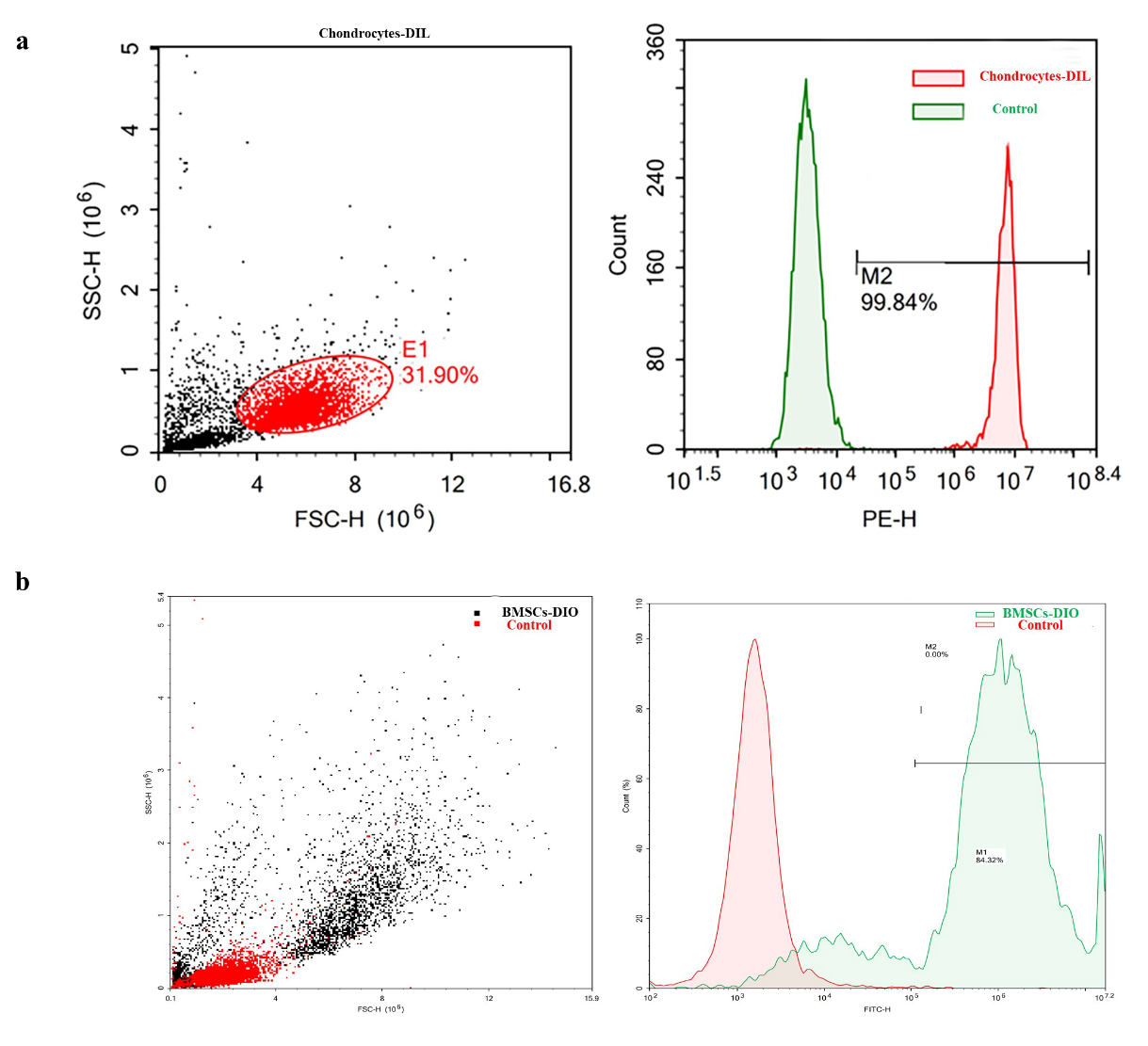 |
| --- |
| **Fig. S3**  Labelling rate detection of cell fluorescent probes by flow cytometry.  a) 99.84% labelling rate of chondrocytes with a red fluorescent probe (DIL). b) 84.32% labelling rate of BMSCs with a green fluorescent probe (DIO). |
| **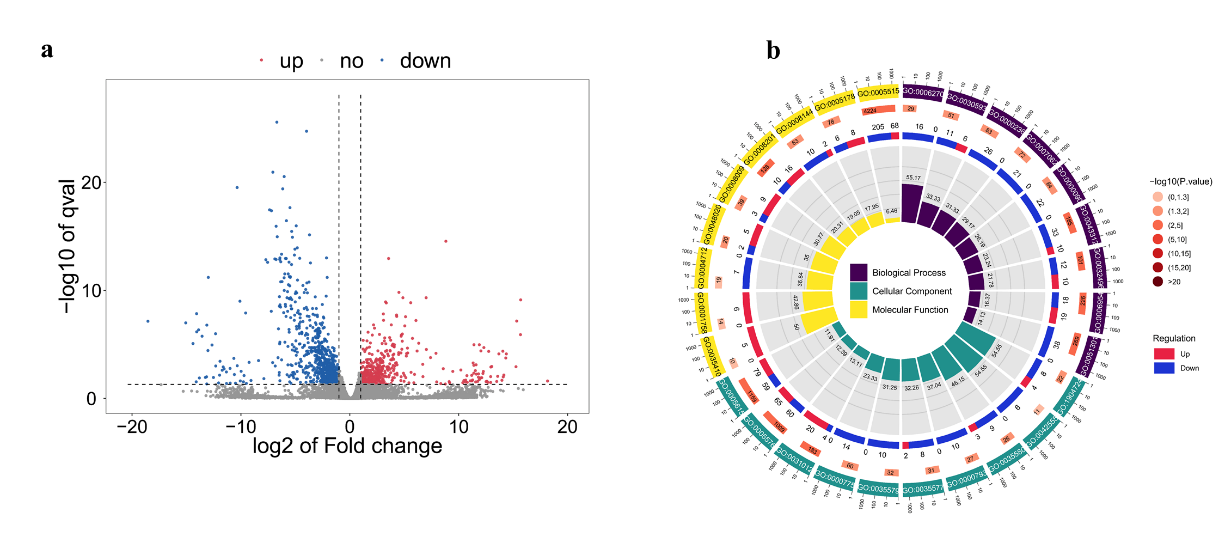** |
| **Fig. S4**  mRNA-seq analysis.  a) The overall distribution of differentially expressed genes in osteochondral samples from the control and E groups after 4.5 months is shown. Red represents significantly up-regulated differentially expressed genes, blue represents significantly down-regulated differentially expressed genes, and grey dots represent non-significantly differentially expressed genes. b) Compared to the control groups, the E groups have 968 differentially expressed genes, including 340 highly expressed genes and 628 low-expressed genes. |
| **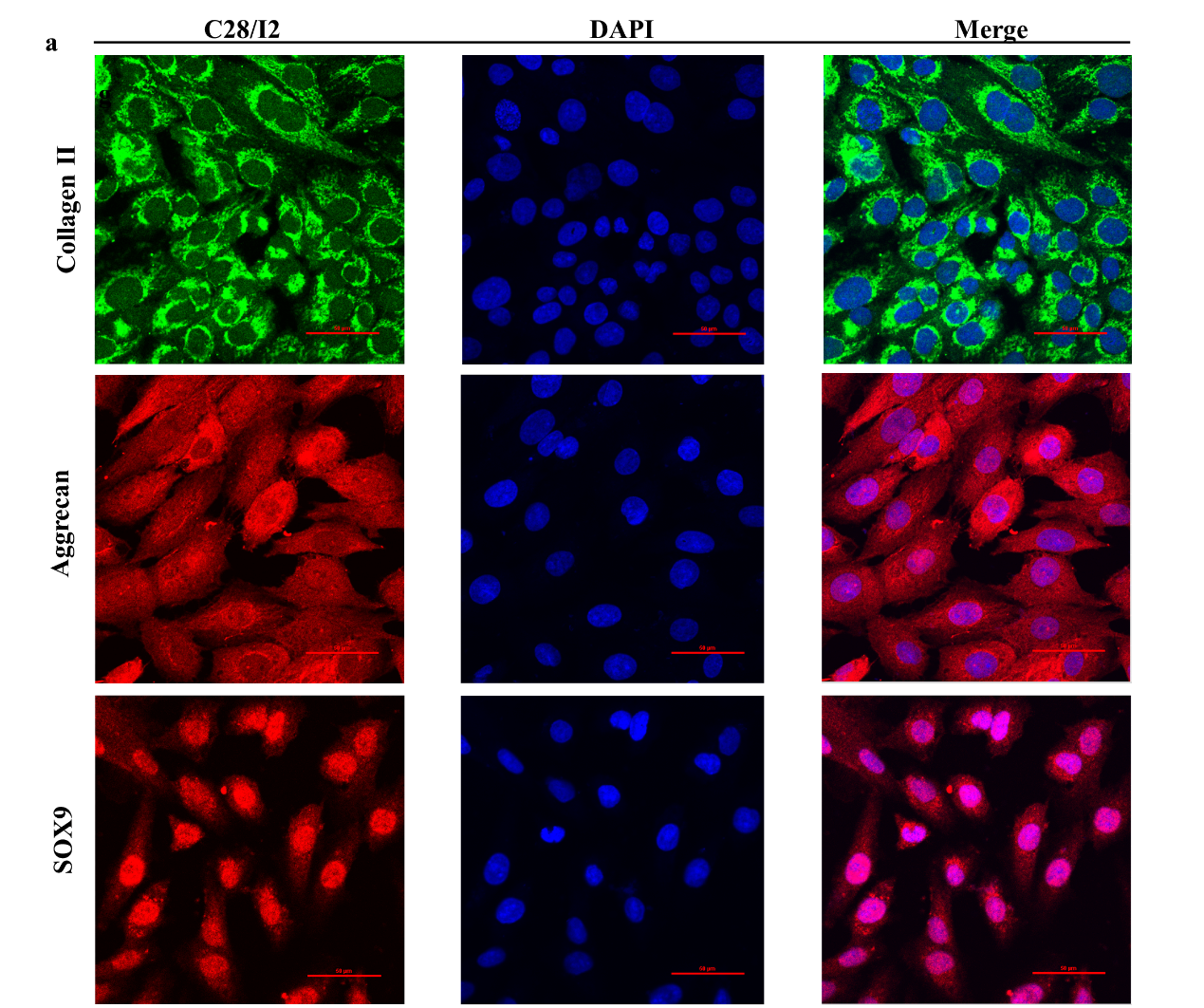** |
| **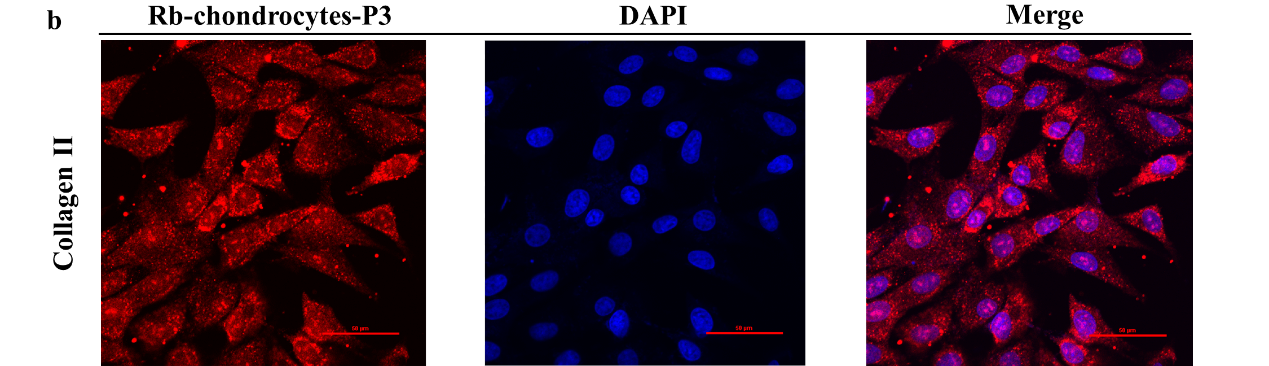** |
| **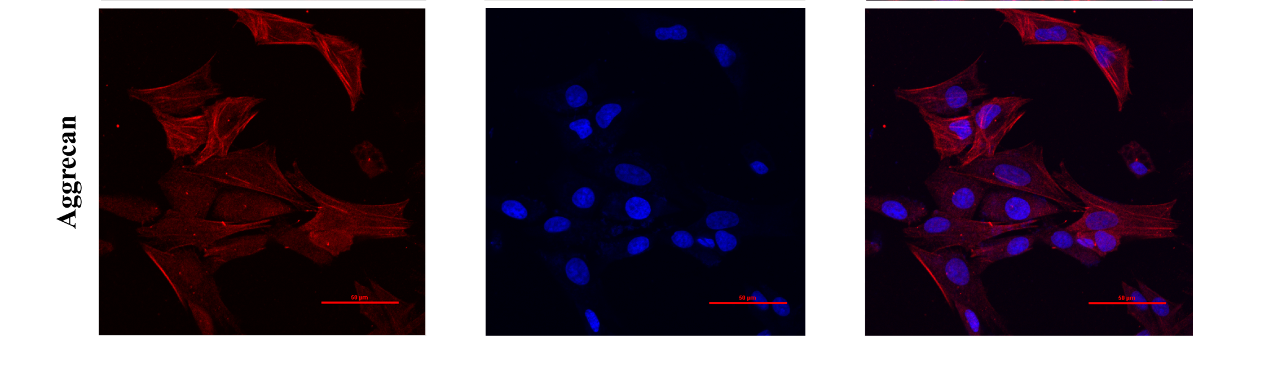** |
| **Fig. S5**  Cell identification.  a) Immunofluorescence (IF) staining of collagen type II, aggrecan, and SOX9 of the human chondrocyte line C28/I2. b) IF staining of collagen type II and aggrecan of rabbit chondrocytes from the knee joint. |
